# Prognostic Significance of Fluorine-18 Fluorodeoxyglucose Positron Emission Tomography in Anal Squamous Cell Carcinoma: A Systematic Review and a Meta-Analysis

**DOI:** 10.1155/2018/9760492

**Published:** 2018-12-04

**Authors:** Ramin Sadeghi, Sara Harsini, Mohammad Ali Qodsi Rad, Vahid Reza Dabbagh, Giorgio Treglia

**Affiliations:** ^1^Nuclear Medicine Research Center, Mashhad University of Medical Sciences, Mashhad, Iran; ^2^Association of Nuclear Medicine and Molecular Imaging (ANMMI), Universal Scientific Education and Research Network (USERN), Tehran, Iran; ^3^Students' Scientific Research Center (SSRC), Tehran University of Medical Sciences, Tehran, Iran; ^4^Research Center for Nuclear Medicine, Dr. Shariati Hospital, Tehran University of Medical Sciences, Tehran, Iran; ^5^Nuclear Medicine Department, Shohadaye Tajrish Hospital, School of Medicine, Shahid Beheshti University of Medical Sciences, Tehran, Iran; ^6^Clinic of Nuclear Medicine and PET/CT Center, Ente Ospedaliero Cantonale, Oncology Institute of Southern Switzerland, Bellinzona, Switzerland; ^7^Health Technology Assessment Unit, Ente Ospedaliero Cantonale, Bellinzona, Switzerland; ^8^Department of Nuclear Medicine and Molecular Imaging, Lausanne University Hospital, Lausanne, Switzerland

## Abstract

**Purpose:**

Prognostic significance of fluorine-18 fluorodeoxyglucose positron emission tomography (^18^F-FDG-PET) in anal squamous cell carcinoma (SCC) has been evaluated in several studies; however, the results seem to be controversial and no consensus exists about its predictive capability. The current meta-analysis was carried out to comprehensively investigate the prognostic significance of ^18^F-FDG-PET parameters in patients with anal SCC.

**Methods:**

A comprehensive literature search of PubMed/MEDLINE and Scopus databases was performed to retrieve pertinent articles published until August 5th 2018, concerning the prognostic significance of ^18^F-FDG-PET in patients with anal SCC. No language restriction was used. Several prognostic factors were reported for progression-free survival (PFS) and overall survival (OS) including pretreatment maximum standardized uptake value (SUVmax), metabolic tumor volume (MTV), inguinal nodal uptake, and metabolic response to therapy.

**Results:**

Eleven studies (741 patients) were included. The pooled hazard ratio (HR) for the probability of PFS was 5.36 (95% confidence interval (95% CI): 3.12–9.21, *p* < 0.001) for metabolic response to therapy and 1.98 (95% CI: 1.26–3.12, *p*=0.003) for SUVmax. The pooled HR for the probability of OS was 5.87 (3.02–11.39, *p* < 0.0001) for metabolic response to therapy. On the other hand, the study revealed that the pooled HRs of MTV and inguinal nodal uptake for PFS were 1.56 (95% CI: 0.96–2.53, *p*=0.072) and 1.79 (95% CI: 1–3.21, *p*=0.051), respectively.

**Conclusions:**

Our findings propose that some ^18^F-FDG-PET parameters could serve as prognostic indicators in anal SCC, but further larger studies are needed in this setting.

## 1. Introduction

Anal carcinomas represent approximately 4% of gastrointestinal cancers diagnosed annually, the majority of which are squamous cell carcinomas (SCCs) [1]. SCC of the anus is an uncommon malignancy but its incidence has increased considerably in recent years among women and men younger than 45 years. Over the past 3 decades, the incidence of anal SCC has increased by approximately 90% in men and 40% in women [2]. The rate of metastatic disease, mostly observed in the liver and the lungs, is low [3]. HIV and human papilloma virus- (HPV-) coinfected patients are at high risk of developing precancerous anal lesions (anal intraepithelial neoplastic lesions) and anal malignancies. Progression and persistence of HPV-associated lesions are known to be enhanced by human immunodeficiency virus- (HIV-) related immunosuppression, which may result in the reactivation of previously acquired HPV infection and loss of control of HPV viral replication [4], the phenomenon which explains the high risk of anal SCC in HIV-infected patients [2].

The diagnosis of anal neoplasms is usually made by physical examination and rectoscopy. In 15–20% of cases, regional lymph node metastases are present at the time of diagnosis [4–6]. Endorectal ultrasound (US) and/or pelvic magnetic resonance imaging (MRI) are required to evaluate tumor depth and regional spread. Haematogenous spread is rare at the time of diagnosis, but 40% of deaths during the course of the disease are due to distant metastases [7]. Anal carcinomas are categorized according to the TNM staging system [5]. The most important prognostic factors for anal cancer are known to be the tumor size and extent (T) and nodal involvement (N) [6]. Response to treatment could also be named as an important prognostic factor for anal cancer [6]. Recently, positron emission tomography with fluorine-18 fluorodeoxyglucose (^18^F-FDG-PET) has become a valuable tool for staging, treatment, and surveillance of patients with various malignancies. Utilization of ^18^F-FDG-PET for accurate staging of anal carcinomas is increasing as a result of several studies performed in this field [8]. There are few reports, however, regarding the utility of ^18^F-FDG-PET as a prognostic indicator in patients with anal cancer.

Abdominoperineal resection (APR) had been the standard method of treatment before 1980 for anal SCC, the mode of therapy which resulted in 5-year recurrence rates of 40–70% and 5-year overall survival (OS) rates of 24–62%. More recent studies, however, indicated that combined chemotherapy and radiotherapy could lead to similar OS rate to surgical treatment [7]. Currently, radiotherapy combined with 5-fluorouracil (5-FU) and mitomycin or 5-FU and cisplatin is used as the standard treatment for anal neoplasms. In the absence of significant toxicity, consolidation chemotherapy is continued either for a predefined time period or until evidence of tumor progression is noted [9]. Surgical treatment is solely considered in relapsed cases or cases with no response to chemoradiotherapy [5, 10]. Adoption of such a therapeutic approach for locally advanced anal SCC has led to a 5-year OS ranging from 61% to 85%. A median OS of only 8–12 months has been reported for those patients with distant metastatic disease or recurrent locally advanced disease not amenable to APR [10].

All the data mentioned above indicates the significance of timely determination of disease recurrence and progression, so as to commence immediate therapeutic approaches, resulting in a better disease prognostication. Prognostic performance of ^18^F-FDG-PET in anal SCC is unclear: several studies have been published over the years on this topic with conflicting results, not reaching a consensus. In addition, a meta-analysis of published studies was missing. The aim of the current investigation is to systematically review and meta-analyze published data about the prognostic performance of ^18^F-FDG-PET in anal SCC in order to provide evidence-based data in this setting.

## 2. Methods

### 2.1. Search Strategy and Study Selection

A comprehensive computer literature search of PubMed/MEDLINE and Scopus databases was carried out to retrieve pertinent published articles concerning the prognostic significance of ^18^F-FDG-PET in patients with anal SCC. We used a search algorithm based on a combination of the terms “(anus OR anal) AND (PET OR positron emission tomography).” No language restriction was used. The search was performed from inception to August 5th, 2018. The bibliographies of eligible studies were also screened to expand our search.

Studies or subsets in studies were included according to the following criteria: (1) more than 5 patients with biopsy-proven anal SCC included; (2) performing at least one ^18^F-FDG-PET scan before and/or after treatment; (3) containing survival data from which the hazard ratio (HR) could be extractable, providing at least one form of survival data, namely, progression-free survival (PFS) or overall survival (OS). Studies investigating the diagnostic role of ^18^F-FDG-PET, in vitro studies and animal experiments, case reports, small case series, review articles, letters, editorials, conference proceedings, commentaries, and articles containing insufficient data to calculate the HRs were excluded. The studies with the most complete or recent data were included in case of data overlap in more studies.

Two researchers independently reviewed titles and abstracts of the retrieved articles, applied the above-mentioned inclusion criteria, rejected ineligible articles, and finally evaluated the full-text version of the included articles to determine their eligibility for inclusion.

### 2.2. Data Extraction and Quality Assessment

Information about basic study characteristics (authors, year of publication, country of origin, study design), patients' characteristics (number of patients with anal SCC performing ^18^F-FDG-PET, median age, gender, TNM staging of the anal SCC, and follow-up time), and technical aspects (injected activity of ^18^F-FDG, acquisition modality, and time interval between ^18^F-FDG administration and scanning) were collected. Furthermore, information about prognostic parameters were extracted including maximum standardized uptake value (SUVmax, calculated as the measure of the greatest amount of ^18^F-FDG uptake in a region of interest divided by body weight), metabolic tumor volume (MTV, determined from the attenuation-corrected PET data using a software), metabolic response to therapy categorized as complete metabolic response (CMR) and partial metabolic response (PMR), inguinal nodal uptake (considering as positive a lymph node with an increased ^18^F-FDG uptake, based on the criteria reported by different authors) and cut off values, as well as the survival data, including PFS and OS with HRs with 95% confidence intervals (95% CIs). Only studies providing such data were finally recruited in the meta-analysis.

Two independent reviewers assessed the methodology of the eligible studies using the Oxford Center for Evidence Based Medicine guideline to examine the quality of prognostic studies [11]. For each included paper, this tool takes into account several parameters comprising patient enrolment at a common point in the course of the disease, follow-up duration, method of verification of outcome, blind outcome assessment of PET findings, and adjustment for important prognostic factors, which could affect the final results [11].

### 2.3. Statistical Analysis

Pooling of HRs and calculation of their 95% CI was performed using a random effects model to determine the prognostic significance of SUVmax, MTV, metabolic response to therapy, and inguinal nodal uptake.

The *I*^2^ statistic was applied to evaluate the heterogeneity among studies, representing the percentage of total variation contributed by a between-study variation and ranging from 0% to 100% [12]. The publication bias was assessed using funnel plots and Egger's regression intercept [13]. All statistical analyses were performed by using Comprehensive Meta-analysis (version 2, Biostat Inc., USA) software. The final results were demonstrated as forest plots.

## 3. Results

### 3.1. Study Characteristics

**Figure 1:**
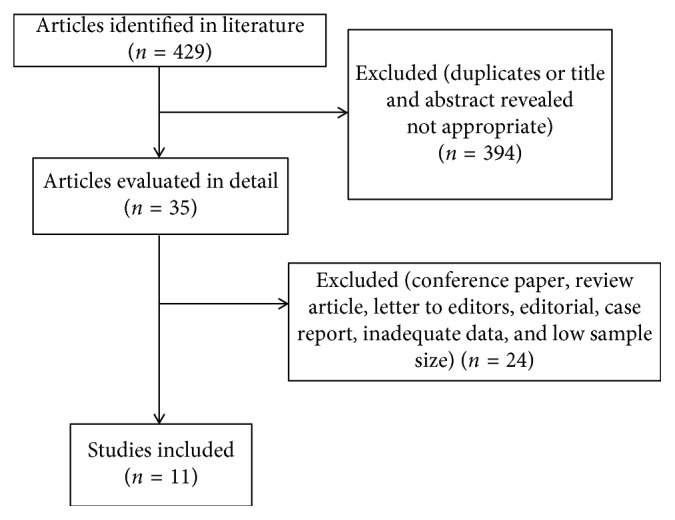
Flow diagram of studies included in the current meta-analysis.

The comprehensive computer literature search from PubMed/MEDLINE and Scopus databases revealed a total of 429 records, among which 394 were excluded after titles and abstracts were screened. The full-texts of the remaining 35 articles were carefully evaluated, and eventually 11 articles (741 patients) [14–24], found to be potentially eligible for inclusion applying the selection criteria mentioned above, were included in the current meta-analysis (Figure 1). No additional studies were retrieved after screening the references of the selected articles. Basic study characteristics and methodological aspects of the 11 retrieved studies are summarized in Tables 1 and 2. As depicted in Table 2, the methodological quality of all included studies has been evaluated according to the Oxford Center for Evidence Based Medicine guideline to examine the quality of prognostic studies [11].

In the current review, five retrospective and six prospective studies about the prognostic significance of ^18^F-FDG-PET in patients with anal SCC have been included.

Table 2 demonstrates all the details regarding the PET prognostic parameters evaluated by each included study. Among the eligible articles, 5 studies (14, 15, 19, 21, and 24) evaluated the prognostic significance of metabolic response to treatment and 3 studies (16, 18, and 23) assessed the prognostic significance of inguinal nodal ^18^F-FDG uptake. The prognostic importance of two other parameters, SUVmax and MTV, has been examined by 5 (14, 17, 18, 20, and 22) and 2 (18 and 20) investigations, respectively.

### 3.2. Pooled Prognostic Significance

**Figure 2:**
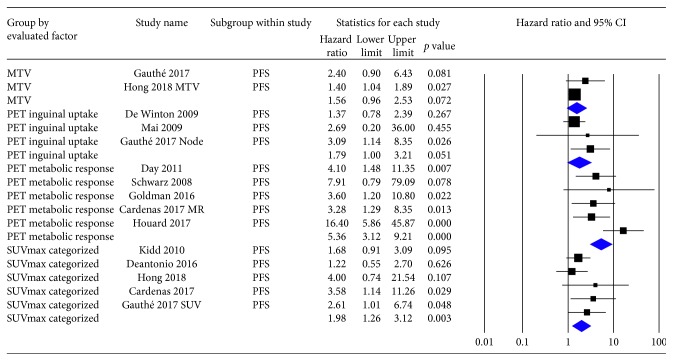
HRs and 95% confidence intervals of individual studies and pooled data of MTV, inguinal nodal ^18^F-FDG uptake, PET metabolic response to therapy, and categorized SUVmax for PFS. HR, hazard ratio; CI, confidence interval; MTV, metabolic tumor volume; PET, positron emission tomography; SUV, standardized uptake value; PFS, progression-free survival.

**Figure 3:**
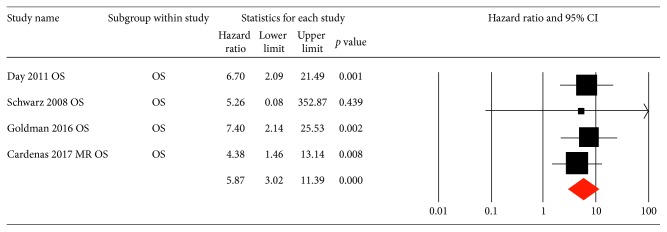
Hazard ratios and 95% confidence interval of individual studies and pooled data of metabolic response for OS. CI, confidence interval; OS, overall survival.

Pooled HRs of MTV, inguinal nodal ^18^F-FDG uptake, metabolic response to therapy, and preoperative SUVmax for PFS were 1.56 (95% CI: 0.96–2.53, *p*=0.07), 1.79 (95% CI: 0.99–3.21, *p*=0.05), 5.36 (95% CI: 3.12–9.21, *p*=0.01), and 1.98 (95% CI: 1.26–3.12, *p* < 0.01), respectively (Figure 2). Four of the eligible studies provided adequate data to perform meta-analysis of the HRs of metabolic response to therapy for overall survival (OS) with a pooled HR of 5.87 (3.02–11.39, *p* < 0.01) (Figure 3).

### 3.3. Heterogeneity and Publication Bias

**Figure 4:**
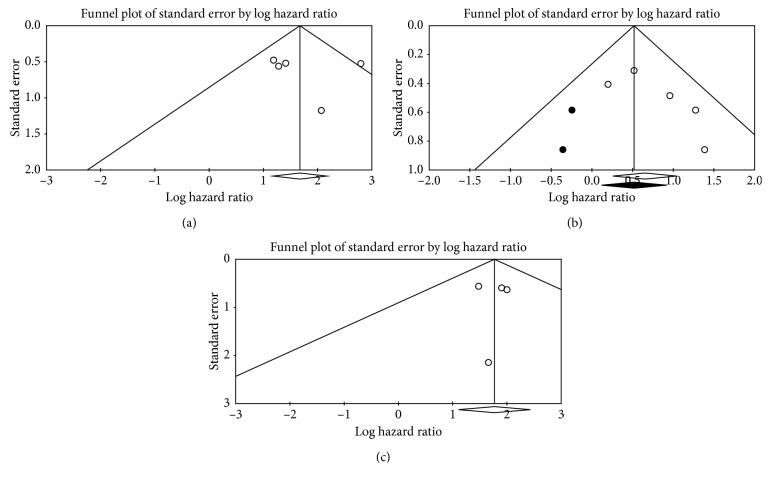
Funnel plots of three meta-analyses of the current study, including metabolic response to therapy for PFS (a) and OS (b), and SUVmax (c).

Few pooled analyses of PET prognostic indices for PFS revealed mild heterogeneity (Table 3). Begg's funnel plot and Egger's test were used to examine the publication bias. The shape of the generated funnel plots seemed asymmetrical, which could signify the presence of possible clinically important publication bias. Then, in order to provide statistical evidence of funnel plot asymmetry, Egger's test was carried out. However, no significant evidence of publication bias of the present meta-analysis (Egger's test *p* values > 0.1) was detected (Figure 4).

## 4. Discussion

^18^F-FDG-PET imaging has been the focus of intensive research, revealing its ever-increasing role in the staging and management of patients with malignant diseases, and this is also the case for anal SCC [8]. Most studies on the role of this imaging modality in anal SCC have focused on its diagnostic and treatment planning significance [25–27]; however, only a few reports exist, analyzing and quantifying the association between PET metabolic parameters and prognosis of anal SCC. As described earlier in this paper, some investigations have indicated the probable roles of certain PET indices, including the SUVmax on pretreatment ^18^F-FDG-PET, MTV, metabolic response as determined by posttherapy ^18^F-FDG-PET (categorized as complete and partial response groups), and inguinal nodal ^18^F-FDG uptake, in yielding prognostic information on either OS or PFS beyond that of established prognostic markers in anal SCC [14–24].

The aforementioned studies on the prognostic value of ^18^F-FDG-PET in patients with anal SCC have mostly revealed somehow contradictory results and could not reach a consensus. The current meta-analysis aimed to examine the prognostic significance of ^18^F-FDG-PET in patients with biopsy-proven anal SCC to provide evidence-based data in this setting. Data from eleven studies (741 patients) were gathered and pooled.

^18^F-FDG-PET may aid in tailoring treatment in patients with anal SCC based on data in the pretreatment and posttreatment settings, providing independently useful clinical information and improving the selection of patients who may benefit from more aggressive treatment [14, 18, 20, 21].

Our meta-analysis demonstrated that metabolic response to therapy and preoperative SUVmax are relevant prognostic factors in patients with anal SCC; therefore, anal SCC patients with inadequate metabolic response to therapy and higher preoperative SUVmax of the anal tumor have a poorer prognosis and they could beneficiate from a more aggressive treatment (such as adequate inguinal irradiation or chemotherapy dose escalation or intensification) that cannot be routinely performed due to the expected increased toxicity [14, 18, 20, 21].

Although the study did not indicate MTV and inguinal nodal ^18^F-FDG uptake as statistically significant prognostic factors, the direction of effect was compatible with other PET metabolic indices. As the number of studies were limited, statistically nonsignificant pooled indices for MTV and inguinal nodal ^18^F-FDG uptake are most likely due to low statistical power.

The current study has some limitations that should be acknowledged when describing the results. Publication bias is a major concern in all meta-analyses as studies reporting significant positive findings are more likely to be published than those reporting negative results. Indeed, it is not unusual for small-sized early studies to report positive findings that subsequent larger studies fail to replicate. We cannot exclude that publication bias may have influenced the results of our analysis.

Furthermore, heterogeneity among studies may represent a potential source of bias in our meta-analysis. This heterogeneity is likely to arise through baseline differences among the patients in the included studies, diversity in methodological aspects between different studies, and different study quality. The overall quality of the studies included in our analysis was not excellent; this was partly caused as a result of lack of patients' recruitment at a common point of the disease course and the inability of the authors of eligible studies to carry out blind outcome assessment to the PET findings. These factors make the overall findings less reliable.

Another limitation to be mentioned is the small number of patients enrolled in some of the included studies, which makes the results of our meta-analysis to be interpreted with caution.

The limited number of investigations evaluating the prognostic significance of ^18^F-FDG-PET parameters can be considered as a limitation. Therefore, larger multicenter studies evaluating the prognostic value of several PET parameters are warranted.

## 5. Conclusions

Our meta-analysis demonstrates that the metabolic response to therapy, detected by ^18^F-FDG-PET, as well as the preoperative SUVmax could serve as promising prognostic markers in patients with biopsy-proven anal SCC. These prognostic markers could indicate which patients may beneficiate from more aggressive treatment. Therefore, ^18^F-FDG-PET may aid in tailoring treatment in patients with anal SCC based on data in the pretreatment and posttreatment settings, providing independently useful clinical information, but further large multicenter studies are needed to strengthen our results.

## Figures and Tables

**Table 1:** Characteristics of selected studies included in the meta-analysis.

First author	Year of publication	Patient source	Number of patients	Median age (range), years	Gender (male/female)	TNM staging	Study design
Schwarz et al. [24]	2008	USA	53^*∗*^	52 (30–89)	20/33	6 stage I, 34 stage II, 8 stage IIIA, and 5 stage IIIB	P
De Winton et al. [16]	2009	Australia	61	57 (27–88)	27/34	19 stage I, 16 stage II, 5 stage IIIA, 19 stage IIIB, and 2 stage IV	P
Mai et al. [23]	2009	Germany	39	59 (37–86)	17/22	9 T1, 21 T2, 5 T3, 4T4, 28 N0, 8 N1, 3 N2	P
Kidd et al. [22]	2010	USA	77^§^	53 (30–89)	33/44	2 stage 0, 7 stage I, 49 stage II, 10 stage IIIA, 9 stage IIIB	P
Day et al. [15]	2011	Australia	48	56 (35–87)	22/26	8 stage I, 18 stage II, 6 stage IIIA, 14 stage IIIB, and 2 stage IV	R
Goldman et al. [19]	2016	USA	148	60 (33–91)	44/104	6 stage I, 64 stage II, 21 stage IIIA, and 58 stage IIIB	R
Deantonio et al. [17]	2016	Italy	55^Ψ^	67 (44–90)	18/38	4 stage I, 25 stage II, 4 stage IIIA, and 22 stage IIIB	P
Gauthé et al. [18]	2017	France	75	63.8 ± 9.9 (40–88)	8/67	5 stage I, 22 stage II, 20 stage IIIA, and 28 stage IIIB	R
Cardenas et al. [14]	2017	USA	110	54.5^€^	48/62	15 stage I, 47 stage II, 48 stage III	R
Houard et al. [21]	2017	France	87	62 (35–89)	19/68	9 T1, 34 T2, 17 T3, 27 T4, 37 N0, 50N+	R
Hong et al. [20]	2018	USA	23	60.1^€^	5/18	3 T1, 8 T2, 9 T3, 3 T4, 13 N0, 10N+	P

^*∗*^The study included 41 cases of squamous cell carcinoma, 8 cases of basaloid carcinoma, 2 cases of adenocarcinoma, 1 case of small cell carcinoma, and 1 case of adenosquamous carcinoma. ^§^The study comprised 65 cases of squamous cell carcinoma, 11 cases of basaloid carcinoma, and 1 case of small cell carcinoma. ^Ψ^The study included 44 cases of squamous cell carcinoma, 3 cases of adenocarcinoma, and 8 cases of cloacogenic carcinoma. ^€^Mean age. P, prospective; R, retrospective.

**Table 2:** Methodological aspects, quality assessment, and main findings of eligible studies.

Study	PET device	Mean FDG dose, MBq	Postinjection interval, min	Quality assessment based on Oxford Center for Evidence-Based Medicine checklist for prognostic studies				PET parameters/cutoff values	Main findings
				Patient enrolment at a common point in the course of the disease	Follow-up duration, months	Method of verification of outcome/blind outcome assessment of PET findings	Adjustment for important prognostic factors		
Schwarz et al. [24]	PET/CT	555–740	40–118	No	5–68 (mean, 26)	Tissue biopsy/NA	Yes	Metabolic response/CMR demarcated as the absence of abnormal FDG uptake at sites of abnormal FDG uptake on the pretreatment FDG-PET study; PMR determined as any persistent abnormal FDG uptake at these sites	CMR in 44 patients, PMR in 9 patients; 2-year CSS of 94% for patients with CMR vs. 39% for patients with PMR (*p*=0.0008); 2-year PFS of 95% for patients with CMR vs. 22% for patients with PMR (*p* < 0.0001); CMR was the most significant predictor of PFS (*p*=0.0003)

De Winton et al. [16]	PET and PET/CT	300–400	≥60	Yes—within 30 days of conventional staging investigations	9–108	Tissue biopsy or radiological progression/yes	Yes	Inguinal nodal FDG uptake/NA	The estimated 5-year OS and PFS for the cohort were 77.3% (95% CI: 55.3–90.4%) and 72.2% (95% CI: 51.5–86.4%), respectively. The estimated 5-year PFS for FDG-PET and conventional imaging staged N2-3 disease was 70% (95% CI: 42.8–87.9%) and 55.3% (95% CI: 23.3–83.4%), respectively

Mai. et al. [23]	PET	266–394	60	No	3–51 (median, 26)	None/yes	Yes	Inguinal nodal FDG uptake/SUVmax > 2.5	No recurrence in inguinal lymph nodes occurred, especially not in patients with CT-enlarged inguinal lymph nodes and elective irradiation only. Patients with PET-positive nodal disease had a higher risk of developing distant metastases (*p*=0.045)

Kidd et al. [22]	PET/CT	555–740	69 ± 21	No	4.9–59.3 (median, 24.2)	Tissue biopsy/no	Yes	SUVmax/NA	Higher SUVmax was associated with worse DFS (*p*=0.05), increased risk of persistent or recurrent disease on posttherapy FDG-PET (*p*=0.0402)

Day et al. [15]	PET and PET/CT	80–120 and 300–400	60	No	20.4–109.2 (median, 60)	Tissue biopsy or radiological progression/no	Yes	Metabolic response/CMR defined as a return of visually graded FDG uptake in all baseline lesions to a level equivalent to or lower than the radioactivity in normal tissues of the involved organ; PMR determined as an improvement in visually graded FDG uptake at baseline involved sites, but persistent residual abnormality	2-year PFS of 95% for patients with a CMR, 71% for PMR, and 0% for NR (*p* < 0.0001); 5-year OS of 88% for CMR, 69% for PMR, and 0% for NR (*p* < 0.0001); metabolic response (CMR versus non-CMR) was a significant prognostic factor: HR for PFS and OS was 4.1 (95% CI: 1.5–11.5, *p*=0.013) and 6.7 (95% CI: 2.1–21.6, *p*=0.002), respectively

Goldman et al. [19]	PET/CT	410.7–851	40–60	No	5–87 (median, 89)	Tissue biopsy and death/no	Yes	Metabolic response/CMR defined as resolution of previously FDG avid primary and/or nodal regions; PMR defined as primary tumors or lymph nodes with persistently abnormal FDG uptake (but decreased compared with pretreatment scan)	2-year PFS for patients with CMR versus non-CMR of 89.8% and 69.2%, respectively (*p*=0.004); 2-year OS for CMR versus non-CMR patients of 94.8% and 79.3% (*p*=0.036)

Deantonio et al. [17]	PET/CT	8^Ψ^	55–90	No	6–66 (median, 51)	Tissue biopsy and radiological progression/no	Yes	SUVmax/NA	PFS and OS were 53% and 77.8% at 2 years and 41.3% and 58.6% at 5 years, respectively, lack of correlation between median SUVmax and clinical response or survival; CMR and T1–T2 stage were statistically significant prognostic factors for PFS (*p* < 0.0001 and *p*=0.02, respectively) and for OS (*p* < 0.0001)

Gauthé et al. [18]	PET/CT	3-4^Ψ^	60	No	10–117 (median, 51)	Tissue biopsy/no	Yes	SUVmax, MTV, inguinal nodal uptake/18, 7 cm^3^, FDG uptake greater than mediastinal uptake, and/or an abnormal anatomical structure on CT greater than 15 mm in shortest diameter, asymmetrically enlarged, or with evidence of central necrosis	Global 4-year OS of 82.7%; significant and independent correlation between MTV at the primary site with OS (*p* < 0.05), as better prognosis was found in patients with MTV less than 7 cm^3^; lack of correlation between SUVmax and survival parameters; correlation of metabolic involvement of the inguinal lymph nodes with a poor outcome in the univariate analysis (*p* < 0.05)

Cardenas et al. [14]	PET/CT	407–740	40–118	No	3.6–94.1 (median, 28.6)	NA/no	Yes	SUVmax, metabolic response/6.1, NA	Significant association between reduced LR and posttreatment SUVmax <6.1 (*p* < 0.0095) and between increased OS and posttreatment SUVmax <6.1 (*p*=0.0086) on univariate analysis; significant association between reduced LR and posttreatment SUVmax <6.1 (*p*=0.0013) and the use of intensity modulated radiation therapy (*p* < 0.001) on multivariate analysis; significant multivariate association between increased OS and posttreatment SUVmax <6.1 (*p* < 0.0373) and the use of chemotherapy (*p*=0.001)

Houard et al. [21]	PET/CT	3.5–4.5^Ψ^	55–90	No	8–76.9 (median, 25)	Tissue biopsy/no	Yes	Metabolic response/CMR defined as the visual absence of pathologic FDG uptake, corresponding to an uptake level equivalent to or lower than that in the normal surrounding organ, PMR determined as any persistent pathologic FDG uptake in the lesions visible at the baseline imaging workup	2-year PFS of 96% for patients with CMR and 28% for non-CMR patients (*p* < 0.0001); 2-year CSS of 100% for patients with CMR and 59% for those without CMR (*p* < 0.0001); CMR was the only significant predictor of PFS and CSS (*p* < 0.0001)

Hong. et al. [20]	PET/CT	296–555	60	PET/CT acquired at radiotherapy simulation and subsequently after chemoradiation therapy	11.76–49.2 (median, 30)	NA/yes	Yes	SUVmax, MTV/NA, NA	Association of pretreatment MTV (HR: 1.4. 95% CI: 1.02–2.05), interim MTV (HR: 1.4, 95% CI: 1.04–1.89), and interim TLG (HR: 1.1, 95% CI: 1.01–1.21) with FFLR

^Ψ^The unit is MBq/kg. HR, hazard ratio; CMR, complete metabolic response; PMR, partial metabolic response; NA, not available; NR, no response; CI, confidence interval; PET, positron emission tomography; CT, computed tomography; SUVmax, maximum standardized uptake value; FDG, fluorine-18 fluorodeoxyglucose; MTV, metabolic tumor volume; TLG, total lesion glycolysis; FFLR, freedom from local and regional recurrence; LR, local recurrence; PFS, progression-free survival; OS, overall survival; CSS, cause-specific survival; DFS, disease-free survival.

**Table 3:** Pooled data of MTV, inguinal nodal ^18^F-FDG uptake, metabolic response, and categorized SUVmax for PFS and OS.

	HR (95% CI)	Overall effect, *p* value	Heterogeneity (d.f.)
*PFS*			
MTV	1.56 (0.96, 2.53)	*Z* = 1.80, *p*=0.07	*I* ^2^ = 5.62% [1]
Inguinal nodal uptake	1.79 (0.99, 3.21)	*Z* = 1.95, *p*=0.05	*I* ^2^ = 3.65% [2]
Metabolic response	5.36 (3.12, 9.21)	*Z* = 6.09, *p*=0.00	*I* ^2^ = 38.31% [4]
Categorized SUVmax	1.98 (1.26, 3.12)	*Z* = 2.95, *p*=0.00	*I* ^2^ = 0% [4]

*OS*			
Metabolic response	5.87 (3.02, 11.39)	*Z* = 5.23, *p*=0.00	*I* ^2^ = 0% [3]

HR, hazard ratio; d.f., degrees of freedom; PFS, progression free survival; MTV, metabolic tumor volume; SUVmax, maximum standardized uptake value; OS, overall survival.
